# Correlation between 12α-hydroxylated bile acids and insulin secretion during glucose tolerance tests in rats fed a high-fat and high-sucrose diet

**DOI:** 10.1186/s12944-020-1193-2

**Published:** 2020-01-15

**Authors:** Reika Yoshitsugu, Keidai Kikuchi, Shota Hori, Hitoshi Iwaya, Masahito Hagio, Hidehisa Shimizu, Tohru Hira, Satoshi Ishizuka

**Affiliations:** 1 Research Faculty of Agriculture, Hokkaido University, Kita-9, Nishi-9, Kita-ku, Sapporo, 060-8589 Japan; 2 Institute of Life and Environmental Science, Academic Assembly, Shimane University, Matsue, 690-8504 Japan

**Keywords:** Energy intake, Enterohepatic circulation, Glucose tolerance, Multiple regression analysis

## Abstract

**Background:**

Previously, we found a significant relationship in a rat study between energy intake and bile acid (BA) metabolism especially 12α-hydroxylated (12αOH) BAs. The present study was designed to reveal relationships among BA metabolism, glucose tolerance, and cecal organic acids in rats fed a high-fat and high-sucrose diet (HFS) by using multivariate and multiple regression analyses in two types of glucose tolerance tests (GTTs).

**Methods:**

Male WKAH/HkmSlc rats were fed with a control or a HFS for 13 weeks. Oral glucose tolerance test (OGTT) and intraperitoneal glucose tolerance test (IPGTT) were performed at week 9 and 11, respectively. BAs were analyzed by using ultra high-performance liquid chromatography-mass spectrometry. Organic acid concentrations in cecal contents were analyzed by using ultra high-performance liquid chromatography with post-column pH buffered electric conductivity method.

**Results:**

A positive correlation of aortic 12αOH BA concentration was observed with energy intake and visceral adipose tissue weight. We found that an increase of 12αOH BAs in enterohepatic circulation, intestinal contents and feces in the HFS-fed rats compared to those in control rats regardless of no significant increase of total BA concentration in the feces in the test period. Fecal 12αOH BA concentration was positively correlated with maximal insulin level in OGTT and area under curve of insulin in IPGTT. There was a positive correlation between aortic 12αOH BAs concentration and changes in plasma glucose level in both OGTT and IPGTT. In contrast, a decrease in the concentration of organic acids was observed in the cecal contents of the HFS-fed rats. Multiple linear regression analysis in the IPGTT revealed that the concentrations of aortic 12αOH BA and cecal acetic acid were the predictors of insulin secretion. Moreover, there was a positive correlation between concentration of portal 12αOH BAs and change in insulin concentration of peripheral blood in the IPGTT.

**Conclusion:**

The distribution analysis of BA compositions accompanied by GTTs revealed a close relationship between 12αOH BA metabolism and insulin secretion in GTTs in rats.

## Background

Non-communicable diseases (NCDs) includes cardiovascular disease, diabetes, cancer and chronic respiratory diseases [1]. Such NCDs are leading cause of death worldwide and the prevalence of NCDs is being increased [2]. Some behavioral factors including harmful use of alcohol, smoking tobacco, physical inactivity and unhealthy diet participate in development of NCDs [1], which suggest that dietary intervention is available to prevent NCDs. One of the devastating disorders is type 2 diabetes mellitus (T2DM) which is characterized as insulin resistance [3]. Recently, association is reported between insulin resistance and bile acid (BA) metabolism, especially 12α-hydroxylated (12αOH) BAs in humans [4, 5].

BA is one of the products in cholesterol catabolism [6] and structure of BAs is based on steroid ring with some hydroxyl groups and a carboxyl group, indicating amphipathic nature of the molecules, which contributes to lipid absorption in the upper small intestine [7] via bile secretion from the liver. In other words, an increase in BA concentration in the gut reflects energy intake in the animal body. BAs are usually reused via uptake by ileal epithelial cells that express sodium-dependent BA transporter [8] in the process referred to as enterohepatic circulation [6]. Since absorbed BAs in enterocytes are released into portal blood and incorporated in hepatocytes, BA concentration is considered to be higher in portal blood than in systemic blood. Those suggest BAs as a marker of energy intake in the body especially for organs related with enterohepatic circulation. Such alteration of BAs in an excess energy retention also can be detected in feces and blood.

A high energy intake not only increases a risk of diabetes but also induces alteration of BA metabolism. We observed that an increase in fecal concentration of 12αOH BAs is positively associated with glucose intolerance in rats fed a HF diet [9]. Such increase in the concentration of 12αOH BAs in a high energy intake might be involved in some early events in development of NCDs including T2DM. A HF and high-sucrose (HFS) diet is an energy-dense diet that includes a large amount of sucrose and also is a model at an early phase of T2DM [3]. To understand association between precise BA metabolism and glucose, we investigated alteration of biochemical parameters that reflect glucose tolerance in rats fed a HFS diet to identify significant relationships among those in an early phase of diabetes.

## Materials and methods

### Animals

Wistar King A Hokkaido male rats (WKAH/HkmSlc) (3-week-old, Japan SLC, Shizuoka, Japan NBRP Rat No: 0154), an inbred strain of Wistar rats, were housed in an air-conditioned room at 22 ± 2 °C temperature with 55 ± 5% humidity, and the light period was from 8:00 to 20:00. The rats were housed individually in wire-bottomed cages and allowed ad libitum access to diet and water, except for the periods performing glucose tolerance tests (GTT). Body weight and food consumption were measured every 2 days. Food consumption was calculated with weight difference of a feeding tray before and after feeding. The rats were acclimatized with a control diet based on the AIN-93G formulation (Table 1) [10]. The acclimatized rats (*n* = 22) were then divided into two groups and fed either the control (C; *n* = 10) or a HFS diet (*n* = 12) for 13 weeks. Tail vein blood was collected from the rats in every 2 week from week 1 to 11 during the test period after food deprivation for 16 h to measure fasting plasma glucose concentration. Also, the feces were collected every 2 week from week 2 to 12 and in week 13 for measuring fecal BA compositions. At the end of the test period, bile juice was collected through a polyvinyl catheter (SV-35; id 0.5 mm, od 0.9 mm; Natsume Seisakusyo, Tokyo, Japan) inserted into common bile-pancreatic duct under anesthesia with sodium pentobarbital (Somnopentyl, 50 mg/kg body weight, Kyoritsu Seiyaku Corporation, Tokyo, Japan). The bile collection was performed for 10 min and the bile flow was measured. Immediately after the bile collection, the blood was collected from portal vein and aorta abdominalis, respectively. An anticoagulant reagent (heparin, 50 U/mL blood, Nakarai Tesque, Inc., Kyoto, Japan) and a protease inhibitor (aprotinin, 500 KIU/mL blood, Fujifilm Wako Pure Chemical Corporation, Osaka, Japan) were added to the collected blood and the plasma was separated. The rats were killed by exsanguination thereafter. The liver, visceral adipose tissues, and intestinal contents were collected and weighed.

**Table 1:** Diet compositions

	C	HFS
	g/kg diet	
Dextrin^a^	529.5	–
Casein^b^	200.0	200.0
Sucrose^c^	100.0	399.5
Soybean oil^d^	70.0	70.0
Lard	–	230.0
Cellulose^e^	50.0	50.0
Mineral mixture^f^	35.0	35.0
Vitamin mixture^g^	10.0	10.0
Choline bitartrate^h^	2.5	2.5
L-Cystine^h^	3.0	3.0

^a^ TK-16 (Matsutani Chemical Industry Co., Ltd., Hyogo, Japan)

^b^ NZMP Acid Casein (Fonterra Co-Operative Group Limited, Auckland, New Zealand)

^c^ Nippon Beet Sugar Manufacturing Co.,Ltd., Tokyo, Japan

^d^ J-Oil Mills, Inc., Tokyo, Japan

^e^ Crystalline cellulose (Ceolus PH-102, Asahi Kasei Corp., Tokyo, Japan)

^f^ AIN-93G mineral mixture [10]

^g^ AIN-93 vitamin mixture [10]

^h^ Fujifilm Wako Pure Chemical Corporation, Osaka, Japan

### BA analysis

The amount of each BA in the bile, intestinal contents (jejunum, ileum, and cecum), and feces were measured after extraction as previously reported [9, 11, 12]. The individual BA concentrations were measured with nordeoxycholic acid (23-nor-5β-cholanic acid-3α,12α-diol) as an internal standard.

12αOH BAs measured in this experiment were as follows: cholic acid (CA; 5β-cholanic acid-3α,7α,12α-triol), deoxycholic acid (DCA; 5β-cholanic acid-3α,12α-diol), ursocholic acid (UCA; 5β-cholanic acid-3α,7β,12α-triol), taurocholic acid (TCA; 5β-cholanic acid-3α,7α,12α-triol-*N*-(2-sulfoethyl)-amide), taurodeoxycholic acid (TDCA; 5α-cholanic acid-3α,12α-diol-*N*-(2-sulfoethyl)-amide,), glycocholic acid (GCA; 5β-cholanic acid-3α,7α,12α-triol-*N*-(carboxymethyl)-amide), glycodeoxycholic acid (5β-cholanic acid-3α,12α-diol-*N*-(carboxymethyl)-amide), 7-oxo-deoxycholic acid (7oDCA; 5β-cholanic acid-3α,12α-diol-7-one), 12-oxo-lithocholic acid (12oLCA; 5β-cholanic acid-3α-ol-12-one), and 5β-cholanic acid-12α-ol-3-one (3o12α).

Non-12αOH BAs measured in this experiment were as follows: α-muricholic acid (αMCA; 5β-cholanic acid-3α,6β,7α-triol), β-muricholic acid (βMCA; 5β-cholanic acid-3α,6β, 7β-triol), ω-muricholic acid (ωMCA; 5β-cholanic acid-3α,6α, 7β-triol), chenodeoxycholic acid (CDCA; 5β-cholanic acid-3α,7α-diol), hyocholic acid (HCA; 5β-cholanic acid-3α,6α, 7α-triol), hyodeoxycholic acid (HDCA; 5β-cholanic acid-3α,6α-diol), ursodeoxycholic acid (UDCA; 5β-cholanic acid-3α, 7β-diol), lithocholic acid (LCA; 5β-cholanic acid-3α-ol), tauro-α-muricholic acid (TαMCA; 5β-cholanic acid-3α,6β,7α-triol-*N*-(2-sulfoethyl)-amide), tauro-β-muricholic acid (TβMCA; 5β-cholanic acid-3α,6β,7β-triol-*N*-(2-sulfoethyl)-amide), tauro-ω-muricholic acid (TωMCA; 5β-cholanic acid-3α,6α,7β-triol-*N*-(2-sulfoethyl)-amide), taurochenodeoxycholic acid (TCDCA; 5β-cholanic acid-3α, 7α-diol-*N*-(2-sulfoethyl)-amide), taurohyodeoxycholic acid (5β-cholanic acid-3α,6α-diol-*N*-(2-sulfoethyl)-amide), taurolithocholic acid (5β-cholanic acid-3α-ol-*N*-(2-sulfoethyl)-amide), glycochenodeoxycholic acid (5β-cholanic acid-3α,7α-diol-*N*-(carboxymethyl)-amide), glycohyodeoxycholic acid (5β-cholanic acid-3α,6α-diol-*N*-(carboxymethyl)-amide), glycoursodeoxycholic acid (5β-cholanic acid-3α,7β-diol-*N*-(carboxymethyl)-amide), glycolithocholic acid (5α-cholanic acid-3α-ol-*N*-(carboxymethyl)-amide), and 7-oxo-lithocholic acid (7oLCA; 5β-cholanic acid-3α-ol-7-one).

All BAs, with the exception of UCA, were purchased from Steraloids, Inc. (Newport, RI, USA); UCA was obtained from Toronto Research Chemicals (Toronto, Ontario, Canada).

### Oral and Intraperitoneal glucose tolerance tests

We performed two types of GTT with oral or intraperitoneal administrations [13] at week 9 and 11, respectively. IPGTT was performed to observe direct response to glucose without influence of its absorption in the intestine. The rats were fasted for 16 h beforehand and fasting blood was collected from the tail vein. A glucose solution was administered orally (OGTT; 2 g/kg) at week 9 or intraperitoneally (IPGTT; 1 g/kg) at week 11. At the indicated time points after glucose injection, blood samples were collected from the tail vein into tubes containing heparin and aprotinin as mentioned above. The plasma was separated by centrifugation and stored at − 80 °C until analysis. The plasma glucose and insulin concentrations were analyzed using Glucose CII-test kit (Wako) and Rat Insulin ELISA kit (AKRIN-010 T) (Fujifilm Wako Shibayagi Coporation, Shibukawa, Japan), respectively. Area under curve (AUC) in plasma glucose and insulin was calculated with the trapezoidal rule in each GTT. For calculation of ΔAUC, the glucose or insulin concentration at the starting point was set to 0. Then, the changes in blood glucose or insulin concentration was plotted in every time point and ΔAUC was from those values.

### Organic acid analysis in cecal contents

Organic acids in the cecal contents were measured by using HPLC (Shimadzu Corporation, Kyoto, Japan) with crotonic acid (Fujifilm Wako Pure Chemical Corportion) as an internal standard [14]. Briefly, the cecal contents were homogenized and neutralized with sodium hydroxide. The hydrophobic substances in the supernatant were removed by chloroform, and the aqueous phase was passed through a membrane filter (cellulose acetate, 0.20 μm pore size; DISMIC-13cp; Toyo Roshi Kaisha, Ltd., Tokyo, Japan). The samples were analyzed by HPLC (Shimadzu Corporation) equipped with a solvent delivery system (SCL-10AVP; Shimadzu Corporation), a double ion-exchange column (Shim-Pack SCR-102H, 8 mm × 300 mm; Shimadzu), and an electro-conductivity detector (CDD-6A; Shimadzu Corporation). The mobile phase was 5 mM of *p*-toluenesulfonic acid, and the detection solution was 5 mM of *p*-toluenesulfonic acid containing 100 μM of EDTA and 20 mM of Bis-Tris.

### Statistical analyses

The significance of differences between C and HFS groups was determined using Student’s *t*-test. Multivariate correlation analysis was performed in each GTT test. Multiple linear regression was performed to determine predictors in various parameters to insulin ΔAUC during the IPGTT. Pearson’s method was used to evaluate simple regression. A *P* value < 0.05 was considered to be significant. JMP version 14.0.0 (SAS Institute Inc., Cary, NC, USA) was used for the statistical analyses.

## Results

There was a decrease in diet consumption in the HFS-fed rats (Table 2) presumably due to energy density. On the other hand, total energy intake was significantly higher in the HFS-fed rats than in C. The body weight in the HFS-fed rats was greater than that in C accompanied by an increased weight of visceral adipose tissues along with the energy intake. There was a significant increase in relative liver weight in the HFS-fed rats than in C-fed rats (*P* < 0.05). Consumption of the HFS diet enhanced basal secretion of the bile juice from the liver.

**Table 2:** Food intake, growth, organ weights and bile flow

	C	HFS
Cumulative food intake		
(g)	1446 ± 30	1225 ± 17*
(kcal)	5707 ± 121	6244 ± 88*
Final body weight (g)	372.4 ± 11.5	417.3 ± 8.7*
Organ weight (g/100 g body weight)		
Liver	2.97 ± 0.05	3.19 ± 0.04*
Visceral fat	7.68 ± 0.22	8.94 ± 0.14*
Mesenteric adipose tissue	1.49 ± 0.07	1.85 ± 0.05*
Epididymal adipose tissue	2.66 ± 0.07	2.76 ± 0.09
Retroperitoneal adipose tissue	2.67 ± 0.12	3.52 ± 0.08*
Perirenal adipose tissue	0.85 ± 0.03	0.81 ± 0.02
Bile flow (μL/min)	12.9 ± 0.33	15.8 ± 1.09*

* Significant difference from the values in C (Student’s *t*-test, *P* < 0.05, *n* = 10 for C, *n* = 12 for HFS)

**Fig. 1:**
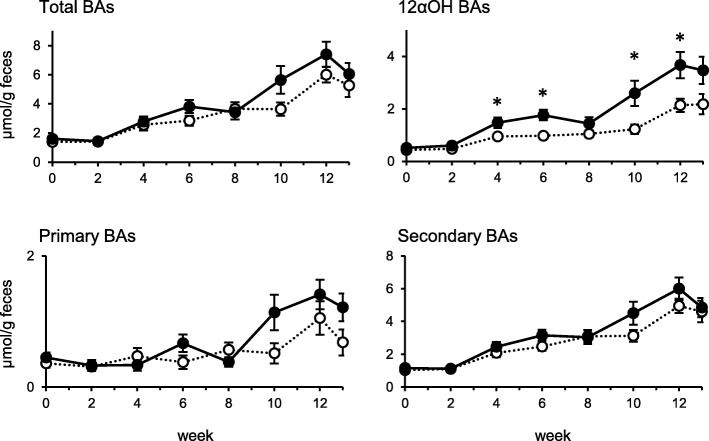
Changes in BA profiles in feces of the rats fed C and HFS diet Feces were collected throughout one day in every two weeks. The BA concentration was shown as total, 12αOH, primary, and secondary BAs. Values are means with SEM (*n* = 10 for C, *n* = 12 for HFS). Open circles, C; filled circles, HFS. Asterisks indicate significant differences compared to the value in C (*P* < 0.05)

**Fig. 2:**
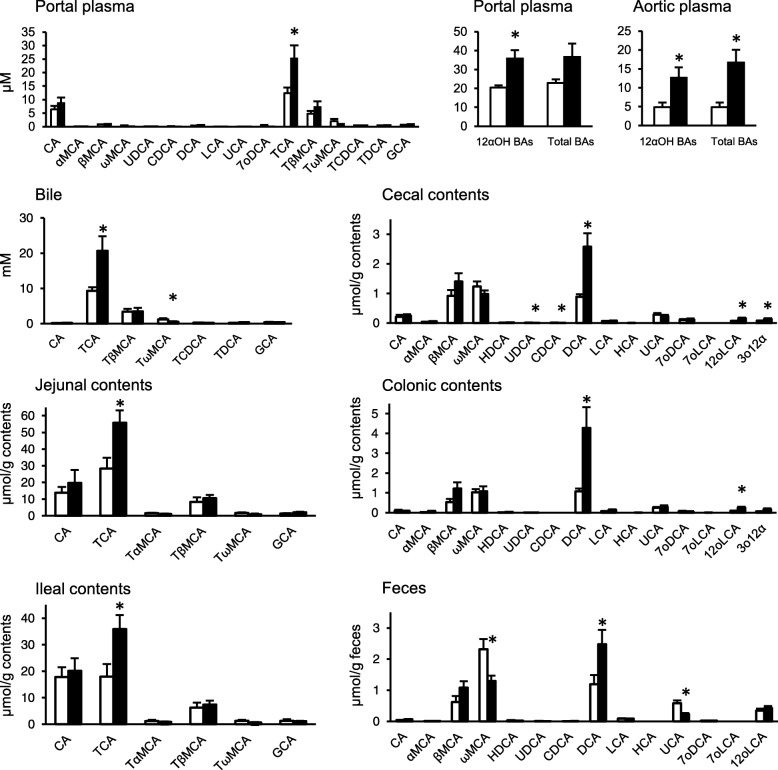
BA compositions in blood, bile, intestinal contents, and feces of the rats fed C and HFS diet. Twenty-nine molecular species of BA was determined in all the samples and the major molecular species were shown in the panels. Values are shown as means with SEM (*n* = 10 for C, *n* = 12 for HFS). Open bars, C; filled bars, HFS. Asterisks indicate significant differences compared to the values in C (*P* < 0.05)

We measured fecal BA concentration during the experiment (Fig. 1). No apparent difference was found in the concentrations of total, primary and secondary BAs. Actual fecal BAs excretion was almost the same because of no significant difference in fecal weight between the groups (data not shown). A significantly higher concentration of 12αOH BAs in feces was observed in the HFS-fed rats at week 4, 6, 10 and 12. In the BA composition at the end of the experiment (Fig. 2), TCA was found to be the major component of BAs in bile juice, small intestine (jejunal and ileal contents) and portal blood especially in the HFS-fed rats. The concentrations of 12αOH BAs and total BAs in aorta blood were lower than that of portal blood. The BA composition in large intestinal contents (cecum and colon) and feces was almost comparable and a high concentration of DCA was found in the large intestinal contents and feces preferentially in the HFS-fed rats. In C, sum of the MCAs (mainly βMCA and ωMCA) was much abundant rather than DCA in the large intestine and feces. In contrast, DCA turned to be the major BA molecule in the HFS-fed rats.

**Fig. 3:**
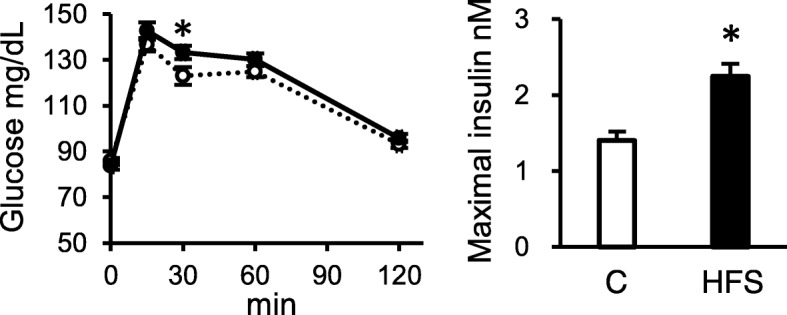
Changes in glucose and insulin concentrations in the blood plasma from tail vein of the rats fed control and HFS diet in oral glucose tolerance test (OGTT) at week 9. After collection of tail vein blood in fasting condition (16 h food deprivation) for time 0, a glucose solution was orally administered (2 g/kg rats) and tail vein blood was collected at the indicated time points to measure glucose. Maximal insulin concentration in blood was determined at 15 min postadministration of glucose. Values are shown as means with SEM (n = 10 for C, n = 12 for HFS). Open circles, C; filled circles, HFS. Asterisks indicate significant differences compared to the values in C (*P* < 0.05)

In the OGTT at weeks 9, the blood glucose was significantly higher at 30 min post administration (Fig. 3) and a significant increase was observed in the glucose ΔAUC in the HFS-fed rats (Table 3). The maximum insulin concentration was higher in the HFS-fed rats than in C-fed rats at 15 min after oral administration of glucose (Fig. 3). Multiple correlation analysis in the parameters in OGTT revealed significant positive correlations of aorta 12αOH BA concentration with energy intake, visceral fat, and glucose ΔAUC (Additional file 1: Table S1). Fecal 12αOH BA concentration was also correlated with the maximal insulin level.

**Table 3:** Parameters in OGTT in rats fed C or HFS at week 9

	C	HFS
Fasting glucose mg/dL	85.7 ± 1.57	83.8 ± 1.67
Glucose AUC mg/dL•h	232.8 ± 3.5	241.6 ± 3.1
Glucose ΔAUC mg/dL•h	60.5 ± 4.4	74.0 ± 3.9*

* Significant difference from the values in C (Student’s *t*-test, *P* < 0.05, *n* = 10 for C, *n* = 12 for HFS)

**Fig. 4:**
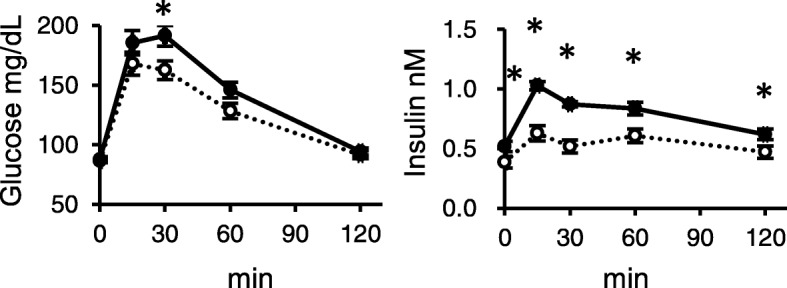
Changes in glucose and insulin concentrations in the blood plasma from tail vein of the rats fed C and HFS diet in intraperitoneal glucose tolerance test (IPGTT) at week 11. After collection of tail vein blood in fasting condition (16 h food deprivation) for time 0, a glucose solution was intraperitoneally injected (1 g/kg rats) and tail vein blood was collected at the indicated time points to measure glucose and insulin concentrations. Values are shown as means with SEM (n = 10 for C, n = 12 for HFS). Open circles, C; filled circles, HFS. Asterisks indicate significant differences compared to the value in C (*P* < 0.05)

In the IPGTT study performed at week 11 (Fig. 4), there was a significant increase of the peak glucose concentration in the HFS-fed rats in response to the glucose injection compared to that in C-fed rats. Also, significant increases were observed in insulin concentration throughout the IPGTT in the HFS-fed rats. Such changes were confirmed in the insulin AUC and the ΔAUC (Table 4). There was an increase in fasting leptin concentration in the HFS-fed rats (Table 4). No difference was observed in the glucose-related parameters, such as fasting glucose, glucose AUC, glucose ΔAUC as well as HOMA-IR between the dietary groups.

**Table 4:** Parameters in IPGTT in rats fed C or HFS at week 11

	C	HFS
Fasting glucose mg/dL	84.1 ± 2.05	89.1 ± 1.99
Glucose AUC mg/dL•h	255.5 ± 9.56	285.5 ± 11.7
Glucose ΔAUC mg/dL•h	82.9 ± 10.3	111.0 ± 11.6
Fasting insulin nM	0.38 ± 0.05	0.53 ± 0.04*
Insulin AUC nM•h	1.09 ± 0.10	1.60 ± 0.06*
Insulin ΔAUC nM•h	0.32 ± 0.06	0.55 ± 0.06*
Relative HOMA-IR	1.00 ± 0.13	1.38 ± 0.13
Fasting leptin μg/mL	120.4 ± 21.7	238.3 ± 20.1*

* Significant difference from the values in C (Student’s *t*-test, *P* < 0.05, *n* = 10 for C, *n* = 12 for HFS)

**Fig. 5:**
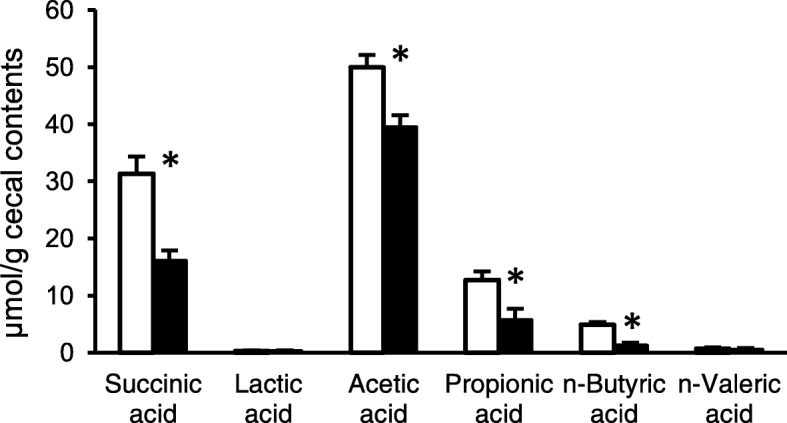
Organic acid concentrations in the cecal contents of the rats fed C and HFS diet. Values are shown as means with SEM (n = 10 for C, n = 12 for HFS). Open circles, C; filled circles, HFS. Asterisks indicate significant differences compared to the value in C (*P* < 0.05)

**Fig. 6:**
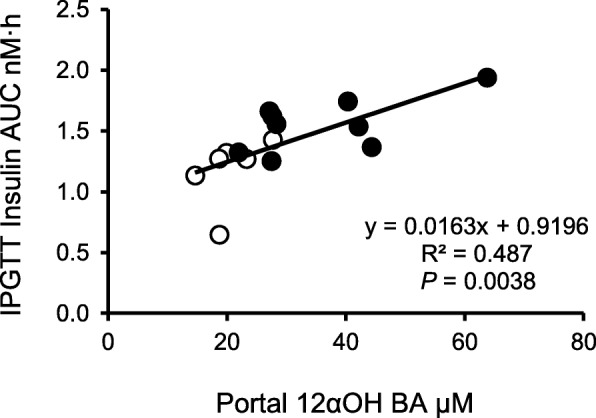
Relationship between concentration of 12αOH BAs in portal plasma and insulin AUC in the IPGTT. Each plot shows individual values. Open circles, C; filled circles, HFS. The insulin AUC was calculated from the data shown in Fig. 4. The portal 12αOH BAs concentration was calculated the data as shown in Fig. 2. Pearson’s method was used to evaluate simple regression

We observed significant decreases in the concentration of succinic acid, acetic acid, propionic acid as well as n-butyric acid (Fig. 5). Multivariate correlation analysis in IPGTT at week 11 (Additional file 2: Table S2) showed that aorta 12αOH BAs at week 13 was positively correlated with energy intake, liver weight, visceral fat, glucose ΔAUC. Fecal 12αOH BAs correlated with insulin ΔAUC, and negatively associated with cecal butyrate level. Multiple linear regression analysis in the IPGTT (Table 5) revealed that changes in aortic 12αOH BA concentration and cecal acetic acid were predictors of insulin ΔAUC in the IPGTT. We also found a significant positive correlation between concentration of 12αOH BAs in portal plasma and insulin AUC in the IPGTT (Fig. 6).

**Table 5:** Predictors of changes in insulin concentration of the rats in IPGTT

Variables	β	*P*-value
Aortic 12αOH BAs	0.46	0.0078
Cecal acetic acid	−0.58	0.0015

Multiple linear regression analysis was performed to determine various parameters to insulin ΔAUC during the IPGTT (*n* = 22). The changes in aortic 12αOH BAs and cecal acetic acid concentration explained 52% of the total variance in the model as predictors of insulin ΔAUC. *R*^*2*^ = 0.568, adjusted *R*^*2*^ = 0.521, *df* = 2, *F* = 11.85, *P* = 0.0005, Durbin-Watson = 1.71

## Discussion

HF feeding in rodents is a diet-induced obese model and displays a variety of disorders such as obesity, insulin resistance and/or dyslipidemia [3]. There is a few paper that describes 12αOH BA metabolism in these experiments. However, an increase in 12αOH BA concentration was observed in tissues related with enterohepatic circulation in rats [9] and there is an association between plasma 12αOH BA concentration and insulin resistance in humans [4]. These observations suggest that 12αOH BA metabolism is involved in alteration of glucose metabolism in these conditions. The present study revealed precise BA metabolism in HFS-fed rats such as significant increase in fecal 12αOH BA concentration in HFS-fed rats regardless of no difference in fecal total BA concentration between the groups. An increase in fecal total BAs might be found if we have much longer experimental period as we observed in our previous experiment [9]. On the other hand, enormous increase was observed in 12αOH BA concentration, especially in bile, small intestinal contents, and portal plasma in the HFS-fed rats, which suggests a selective recycle of 12αOH BAs in enterohepatic circulation in the HFS-fed condition. In contrast, non-12αOH BA seemed to be easily excreted in feces of HFS-fed rats. This study also revealed a significant correlation between portal 12αOH BA concentration and insulin secretion in IPGTT (Fig. 6). These results suggest a close association between enterohepatic 12αOH BAs circulation and glucose metabolism in rats.

As shown in Fig. 2, HFS diet induced selective increase in the concentration of TCA, one of the 12αOH BAs, in the ileal contents and portal blood. TCA is possible to induce insulin secretion from β cells in pancreas via Gpbar1/TGR5 [15–17]. These studies suggest a direct role of TCA in insulin secretion. Since an increase of insulin secretion was observed during OGTT in portal plasma of the rats fed a HFS diet in our previous study [13], it is possible that TCA enhances insulin secretion from β cells. Additionally, TCA induces glucagon-like peptide-1 (GLP-1) secretion via Gpbar1/TGR5 in STC-1 cells, an enteroendocrine cell line [18], suggesting promotion of insulin secretion via GLP-1 from enteroendocrine L-cells. Actually, there was a positive association between 12αOH BAs and insulin secretion in the present study (Fig. 6). Although we did not determine insulin resistance directly, it is possible that the increase in insulin secretion in the HFS-fed rats was partially due to the increase in 12αOH BA concentration in the enterohepatic circulation regardless of insulin sensitivity.

Acetic acid derived from dietary fiber and nondigestible saccharides by fermentation in large intestine can activate 5′-AMP-activated protein kinase (AMPK) [19–21]. Considering AMPK activation contributes to insulin sensitivity [22], those observations suggest that the decrease of acetic acid concentration in the cecal contents of the rats fed with the HFS diet plays a role in insulin tolerance. In the present study, acetic acid in the cecal contents was the predictor of the insulin ΔAUC together with 12αOH BA in aortic plasma by the multiple correlation analysis (Table 5), suggesting that increase in 12αOH BA and reduction in cecal acetic acid concentration contribute to enhancement of insulin secretion.

Several studies show that BA depletion influences glucose homeostasis by BA sequestration (BAS) or abrogation of enzymes responsible for BA production, such as Cyp7a1 and Cyp8b1 as follows. BAS ameliorates glucose homeostasis in *ob*/*ob* mice [23], *db*/*db* mice [24], fatty liver Shionogi mice [25], and also in humans [26]. Those observations suggest that depletion of BAs from intestinal luminal contents improves glucose homeostasis in both humans and mice. In mice, abrogation of Cyp7a1, the rate-limiting enzyme in BA synthesis [27], improves glucose metabolism in mice fed a HF/high-cholesterol diet. Additionally, a dietary supplementation of CA at 0.03% deteriorates glucose metabolism in the Cyp7a1 KO mice. Another report shows that a retarded glucose clearance in mice fed high-cholesterol diet is normalized by abrogation of Cyp8b1, the enzyme responsible for production of 12αOH BAs [28].

Although the present experiment did not allow an increase in the concentration of total BAs in feces of the rats fed HFS diet during the experimental period (Fig. 1), we could detect a significant increase of 12αOH BA concentration in the feces. Such increase in the proportion of 12αOH BAs may be induced by steroidogenic acute regulatory protein (StAR) that transfers cholesterol to mitochondria to produce non-12αOH BAs [29]. The expression of StAR is attenuated in a HF-fed mice and over expression of StAR improves insulin resistance induced by a HF diet [29]. An upregulation of Cyp8b1 in the liver might be another option to enhance the proportion of 12αOH BAs because Cyp8b1 is constitutively expressed in liver [30]. In comparison with our previous experiment in rats fed a HF diet [9], substitution of carbohydrate source from starch to sucrose may not influence the BA metabolism by the lard supplementation in the diet because almost no difference was observed in the BA metabolism of the rats fed either HF [9] or HFS diet (Fig. 2).

Another interesting observation in comparison between the present and our previous studies was a difference in relative liver weights between the HFS-fed rats in the present study and the HF-fed rats in our previous study [9]. As expected, an increase was observed in relative weights of visceral adipose tissues in the rats fed either the HFS or HF diet. On the contrary, the HFS diet increased relative liver weight as compared with that in control (Table 2) whereas the HF diet rather decreased the relative liver weight in our previous study [9]. The difference in the relative liver weight between HFS and HF is considered to depend on carbohydrate source. Obviously, the HFS diet contains nearly 40% of sucrose in the diet that includes 20% of fructose in weight basis in the diet. It is suggested that fructose in the HFS diet seems to be a source of steatosis probably via fatty acid synthesis [31]. The increase in BA concentration in the enterohepatic circulation of a HF-fed rats might have a role in the incorporation of fructose as well as glucose in hepatocytes, resulting in fatty acid synthesis. Such enlargement of liver size might be in relation with hepatic lipid accumulation that deteriorates glucose homeostasis.

## Conclusions

Both 12αOH BAs and cecal acetic acid were predictors for insulin secretion in multiple regression analysis. Additionally, there was a significant positive correlation between portal 12αOH BA concentration and insulin secretion in the IPGTT. These results revealed an association between 12αOH BA metabolism and insulin secretion in GTTs in rats. Modulation of 12αOH BAs and organic acid in the gut might be available to normalize glucose homeostasis in a high-energy consumption.

## Supplementary information

**Additional file 1: Table S1.** Correlation between each pair of variables in growth, OGTT, and 12αOH BAs.

**Additional file 2: Table S2.** Multiple correlations between each pair of variables in growth, IPGTT, 12αOH BAs, and cecal organic acids.

## Data Availability

The datasets analyzed during the current study are included in the manuscript.

## Abbreviations

12oLCA: 12-oxo-lithocholic acid
12αOH: 12α-hydroxylated
3o12α: 5β-cholanic acid-12α-ol-3-one
7oDCA: 7-oxo-deoxycholic acid
7oLCA: 7-oxo-lithocholic acid
AMPK: AMP-activated protein kinase
BA: Bile acid
C: Control
CA: Cholic acid
CDCA: Chenodeoxycholic acid
DCA: Deoxycholic acid
FGF: Fibroblast growth factor
GCA: Glycocholic acid
GLP-1: Glucagon-like peptide-1
GTT: Glucose tolerance test
HCA: Hyocholic acid
HDCA: Hyodeoxycholic acid
HF: High-fat
HFS: High-fat and high-sucrose
IPGTT: Intraperitoneal glucose tolerance test
LCA: Lithocholic acid
MCA: Muricholic acid
NCD: Noncommunicable disease
OGTT: Oral glucose tolerance test
StAR: Steroidogenic acute regulatory protein
T2DM: Type-2 diabetes mellitus
TCA: Taurocholic acid
TCDCA: Taurochenodeoxycholic acid
TDCA: Taurodeoxycholic acid
TαMCA: Tauro-α-muricholic acid
TβMCA: Tauro-β-muricholic acid
TωMCA: Tauro-ω-muricholic acid
UCA: Ursocholic acid
UDCA: Ursodeoxycholic acid
