# Supplementary material for: Correlation between 12α-hydroxylated bile acids and insulin secretion during glucose tolerance tests in rats fed a high-fat and high-sucrose diet
Source: Lipids Health Dis. 2020 Jan 15;19:9. doi: 10.1186/s12944-020-1193-2 (PMC6964016; doi:10.1186/s12944-020-1193-2)
Supplement: Supplementary file 1 — Additional file 1: Table S1. Correlation between each pair of variables in growth, OGTT, and 12αOH BAs. [file 12944_2020_1193_MOESM1_ESM.docx]

Table S1 Correlation between each pair of variables in growth, OGTT, and 12αOH BAs

Variables 1 2 3 4 5 6 7 8

1. Energy intake -

2. Visceral fat 0.8641*** -

3. Fasting glucose 0.1377 -0.0745 -

4. Glucose AUC 0.4651* 0.4754* 0.1216 -

5. Glucose ΔAUC 0.2607 0.4242 -0.6360** 0.6887*** -

6. Maximal insulin 0.6890** 0.5964** 0.1547 0.3915 0.1912 -

7. Fecal 12αOH BAs 0.3584 0.3920 0.3100 0.1069 -0.1418 0.5608* -

8. Aortic 12αOH BAs 0.5878** 0.6269** -0.4330* 0.3588 0.5965** 0.3106 -0.0227 -

Data of aortic 12αOH BAs and fecal 12αOH BAs were obtained at week 13 and week 10, respectively.

*** *P* < 0.001, ** *P* < 0.01, * *P* < 0.05
