# Supplementary material for: Correlation between 12α-hydroxylated bile acids and insulin secretion during glucose tolerance tests in rats fed a high-fat and high-sucrose diet
Source: Lipids Health Dis. 2020 Jan 15;19:9. doi: 10.1186/s12944-020-1193-2 (PMC6964016; doi:10.1186/s12944-020-1193-2)
Supplement: Supplementary file 2 — Additional file 2: Table S2. Multiple correlations between each pair of variables in growth, IPGTT, 12αOH BAs, and cecal organic acids. [file 12944_2020_1193_MOESM2_ESM.docx]

Table S2 Multiple correlations between each pair of variables in growth, IPGTT, 12αOH BAs, and cecal organic acids

Variables 1 2 3 4 5 6 7 8 9 10 11 12 13

1. Energy intake -

2. Liver 0.8044** -

3. Visceral fat 0.8641*** 0.8661*** -

4. Fasting glucose 0.5670* 0.3406 0.4576 -

5. Glucose ΔAUC 0.2605 0.4484 0.3587 -0.2153 -

6. Fasting insulin 0.4658 0.6505** 0.5281* 0.3642 0.0565 -

7. Insulin ΔAUC 0.3793 0.3561 0.3540 -0.0828 0.6074* -0.1472 -

8. Fasting leptin 0.7468** 0.6342** 0.7226** 0.4497 0.0635 0.4711 0.2543 -

9. Fecal 12αOH BAs 0.2786 0.4291 0.2637 -0.1250 0.4899 0.1318 0.5496* 0.2137-

10. Aortic 12αOH BAs 0.6280** 0.6832** 0.6811** 0.2014 0.5907* 0.3050 0.4809 0.4934 0.2764 -

11. Cecal acetic acid -0.3012 -0.2946 -0.2584 0.0299 -0.3844 0.0132 -0.5286* -0.2660 -0.3325 -0.0300 -

12. Cecal butyric acid -0.2675 -0.4342 -0.3079 0.0258 -0.2485 -0.0619 -0.5425* -0.1080 -0.7446** -0.1801 0.6179* -

13. Cecal succinic acid -0.5794* -0.5737* -0.5808* -0.4862 -0.0856 -0.2706 -0.4077 -0.7338** -0.4540 -0.3008 -0.4736 0.3919 -

Data of aortic 12αOH BAs and fecal 12αOH BAs were obtained at week 13 and week 12, respectively. *** *P* < 0.001, ** *P* < 0.01, * *P* < 0.05
